# A carbon balance model for the great dismal swamp ecosystem

**DOI:** 10.1186/s13021-017-0070-4

**Published:** 2017-01-25

**Authors:** Rachel Sleeter, Benjamin M. Sleeter, Brianna Williams, Dianna Hogan, Todd Hawbaker, Zhiliang Zhu

**Affiliations:** 1 Eastern Geographic Science Center, United States Geological Survey, Reston, VA 20192 USA; 2 Western Geographic Science Center, United States Geological Survey, Menlo Park, CA 94025 USA; 3 Geosciences and Environmental Change Science Center, United States Geological Survey, Denver, CO 80225 USA

**Keywords:** Net ecosystem carbon balance, Peatland restoration, Carbon sequestration, Great dismal swamp ecosystem, Lateral west fire, LUCAS model

## Abstract

**Background:**

Carbon storage potential has become an important consideration for land management and planning in the United States. The ability to assess ecosystem carbon balance can help land managers understand the benefits and tradeoffs between different management strategies. This paper demonstrates an application of the Land Use and Carbon Scenario Simulator (LUCAS) model developed for local-scale land management at the Great Dismal Swamp National Wildlife Refuge. We estimate the net ecosystem carbon balance by considering past ecosystem disturbances resulting from storm damage, fire, and land management actions including hydrologic inundation, vegetation clearing, and replanting.

**Results:**

We modeled the annual ecosystem carbon stock and flow rates for the 30-year historic time period of 1985–2015, using age-structured forest growth curves and known data for disturbance events and management activities. The 30-year total net ecosystem production was estimated to be a net sink of 0.97 Tg C. When a hurricane and six historic fire events were considered in the simulation, the Great Dismal Swamp became a net source of 0.89 Tg C. The cumulative above and below-ground carbon loss estimated from the South One and Lateral West fire events totaled 1.70 Tg C, while management activities removed an additional 0.01 Tg C. The carbon loss in below-ground biomass alone totaled 1.38 Tg C, with the balance (0.31 Tg C) coming from above-ground biomass and detritus.

**Conclusions:**

Natural disturbances substantially impact net ecosystem carbon balance in the Great Dismal Swamp. Through alternative management actions such as re-wetting, below-ground biomass loss may have been avoided, resulting in the added carbon storage capacity of 1.38 Tg. Based on two model assumptions used to simulate the peat system, (a burn scar totaling 70 cm in depth, and the soil carbon accumulation rate of 0.36 t C/ha^−1^/year^−1^ for Atlantic white cedar), the total soil carbon loss from the South One and Lateral West fires would take approximately 1740 years to re-amass. Due to the impractical time horizon this presents for land managers, this particular loss is considered permanent. Going forward, the baseline carbon stock and flow parameters presented here will be used as reference conditions to model future scenarios of land management and disturbance.

## Background

Quantifying the impacts of land use, land management, and natural disturbance on terrestrial carbon (C) dynamics is increasingly important as new policies are developed requiring land managers to incorporate scientific information on C storage and flux into the decision making process. Public lands encompass large contiguous regions of forests, rangelands and wetlands in the U.S. and are critical components of the U.S. C balance [1, 2]. The potential to sequester additional C in above-ground vegetation and soils requires a comprehensive analysis of C stocks, flux, while considering key drivers of land-use and natural disturbance [3]. Federal and State policy directives [4–6] have assigned greenhouse gas reduction targets influencing the way in which public lands are monitored, measured and assessed, in order to quantify the benefits of C storage and sequestration. To achieve policy goals, land managers heavily rely on scientific information and models to inform decision-making.

Models can be valuable tools to help simulate and communicate the complex interactions between the key controlling processes of a particular ecosystem; however, challenges associated with model integration, computational constraints, and spatial and temporal data continuity, can compromise model development, validation and magnify uncertainty. Despite the need for simulation models that link land use, management and disturbance with C cycle dynamics, there is a lack of readily available, well-documented, modeling platforms relevant at multiple scales [7]. There are a range of process-based, biogeochemical models, such as CENTURY and Biome BGC [8, 9] that are publicly available, peer-reviewed, incorporate empirical data, and have been applied to many land-use and ecological frameworks. There are fewer examples of land change models that meet the same comprehensive criteria [7] because land change models commonly require data to reflect human behavior, land management preferences, and socio-economic indicators. These data are often unique to local conditions and difficult to scale up or apply to larger regions. For example, agent-based models such as UrbanSim and Swarm [10, 11] use “agents” to calculate the behavior of human actors (e.g. land managers, farmers, developers). These models are designed to answer specific land management questions but are often limited in scalability to other geographic areas. Cellular automata models such as SLEUTH [12] and CLUE-S [13] use transition rules determined by the spatial neighborhood of adjacent cells and have been used for multiple applications, but lack rule-based successional trajectories that may occur after a disturbance.

A new lineage of state-and-transition simulation models (STSM) were developed (i.e. VDDT, ST-Sim and LANDSUM) [14–16], with the underlying purpose of understanding the natural disturbance and succession regime of an ecological system. The STSM architecture uses a non-stationary Markov chain where the probability of a state transitioning to another state can differ at particular time-steps. As a result, complex relationships between landscape behavior and management can be parameterized. STSM’s are similar to cellular automata and Markov chain models, but have been modified to improve simulation rules, spatial patterns, and provide for robust data integration [15, 17]. For a thorough review of STSM models, see Daniel et al. [18]. We use the publicly available ST-Sim software package [19] as the underlying platform for the Land Use and Carbon Scenario Simulator (LUCAS) model. The ST-Sim software platform offers a spatially explicit STSM coupled with a C Stock-Flow model to track annual flows of C as a function of land use, disturbance and management. A combination of stochastic, deterministic and empirical parameters guides the transition from one state to another within a selected time period [18].

**Fig. 1:**
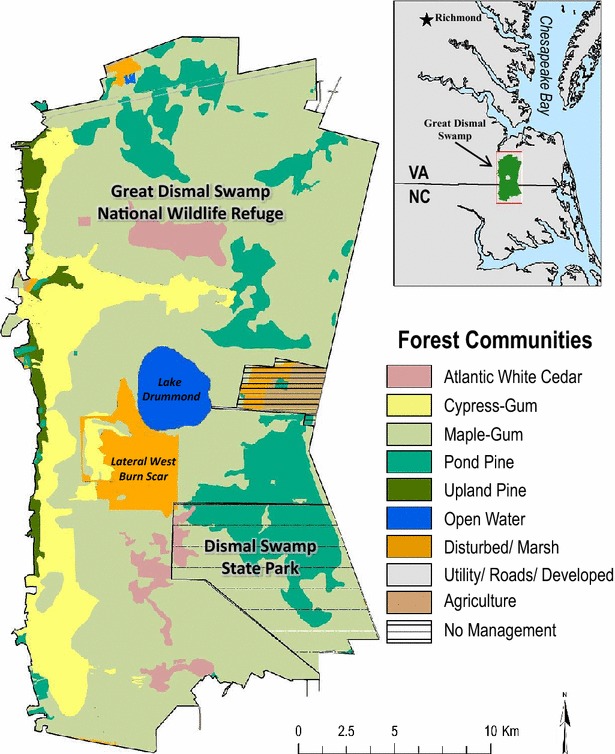
The Great Dismal Swamp study area includes the USFWS National Wildlife Refuge and the Dismal Swamp State Park. Classification of the natural communities in the Great Dismal Swamp follows ‘The Natural Communities of Virginia’ [21]. The study area comprises 54,000 ha. These vegetation communities represent the major forest types included in the carbon balance model and ecosystem services assessment. We model transitions for Atlantic white cedar, cypress-gum, maple-gum and pond pine, but do not model any transitions for the upland pine class

This paper demonstrates an application of the LUCAS model [20] developed for local-level land management at the U.S. Fish and Wildlife Service (USFWS), Great Dismal Swamp (GDS) National Wildlife Refuge in Virginia. The modeling framework is used to estimate the effect of land use, land management, and ecosystem disturbance on C balance in four select vegetation types for the GDS (Fig. 1). To verify and validate the effectiveness of the model application, we characterize annual changes in C stocks and fluxes over a 30-year historical period (1985–2015). We estimate the net ecosystem C balance by considering past ecosystem disturbances resulting from storm damage, fire, and land management actions including hydrologic inundation, vegetation clearing, and replanting. Primarily we focus on two catastrophic fires (South One and Lateral West), and model C loss as an impact from fire disturbance. The GDS application of the LUCAS model has also been developed as a tool to evaluate priority ecosystem services, including C sequestration, as a function of future adaptive management strategies.

### Study area and disturbance history

The GDS study area comprises about 54,000 ha, straddling the state border between Virginia and North Carolina. Two main administrative units encompass the study area—the Great Dismal Swamp National Wildlife Refuge (GDSNWR) and Dismal Swamp State Park in North Carolina (Fig. 1). In the late 18th century, the “Dismal Swamp” was documented by surveyors to be 400,000 ha of undrained wetland; however, after two centuries of draining, ditching, and logging for timber, the swamp was hydrologically and ecologically transformed. In 1974, the GDSNWR was established with the primary purpose of preserving, protecting and restoring the pre-disturbance, native ecosystem. Currently, the USFWS manages the hydrology, forestry, wildlife and fire regimes of the GDSNWR. The complex hydrologic regime is managed by using water control structures to adjust inundation levels within the swamp. Optimal hydrologic conditions are essential for restoring and protecting desired vegetation communities, flood management, and reducing catastrophic fire. Forestry management consists of restoring the once dominant Atlantic white cedar by removing competing species, replanting, and monitoring water levels. Wildlife monitoring is managed through inventory programs of seasonal bird counting. Fire management includes suppression as well as prescribed burns.

To understand and model the vegetation and C dynamics in the GDS, one must gain an understanding of the disturbance regime in the past. The GDS experienced a series of catastrophic disturbances in the historic period from 1985 to 2015. Beginning in 2003, the strong winds from Hurricane Isabel uprooted and flattened approximately 1200 ha of the last pure stands of Atlantic white cedar in the GDS. In response to the impact of the hurricane, the USFWS initiated a large restoration effort to mechanically remove the downed deadwood and replant Atlantic white cedar. With restoration efforts only weeks from completion, and a persistent drought underway, a mechanical spark ignited the South One fire in 2008, lasting 121 days and consuming 2400 ha. Fire consumption included much of the above-ground biomass and a portion of the peat soil. Restoration efforts resumed after the South One fire, but less than 5 years later, lightning ignited the Lateral West fire in 2011. The fire lasted 111 days, burning an estimated 2500 ha, and triggering peat burns up to 1 m deep in some areas. The damage from the second fire caused the forested peat land to transition to standing water and marsh. The biophysical conditions leading up to these disturbances as well as the impacts from the combination of events can be modeled and quantified.

#### Hydric soils

The GDS is a forested wetland, consisting of hydric soils that formed under repeated conditions of saturation, long enough during the growing season to develop anaerobic conditions in the upper 40 cm [22]. Saturation or inundation creates an anoxic environment, fueling microbial activity by the capture of C into the soil. Certain biogeochemical processes, such as the accumulation of organic matter and slow decomposition rates, result in deep peat profiles (0.3–3 m) with relatively low bulk densities ranging from (0.09 to 0.24 cm^3^), and C content ranging from (46 to 64%) [23]. These unique conditions support a limited range of vegetation species, including Atlantic white cedar (*Chamaecyparis thyoide*), bald cypress/tupelo-gum (*Taxodium distichum–Nyssa biflora*), red maple/black gum (*Acer rubrum*–*Nyssa sylvatica*), and pond pine (*Pinus serotina*).

#### Forest communities

Atlantic white cedar has a native geographic range from Maine to Florida and west to the Mississippi [24]. Pure stands of over 50,000 ha once dominated the GDS, yet recent vegetation maps show the species covering about 1600 ha, only 3% of the GDS (Fig. 1). Favorable conditions for Atlantic white cedar are considered stressful for other conifer species. These conditions include 4–6 months annually of water inundation, a shallow water table averaging 10 cm from the surface during the growing season, acidic soils with a pH ranging from 3.2 to 4.4 [24, 25], and fire-dependency for natural re-generation. Atlantic white cedar show steady height and diameter breast height (DBH) growth until 40 or 50 years of age. After 50 years, their height reaches its maximum, but DBH growth continues to increase until 100 years of age [26]. Natural mortality averages between 70 and 200 years (Table 1); however, mortality is greatly influenced by disturbances such as draining, drought, invasion of hardwoods within the first 5 years of growth, fire or hurricane winds. Catastrophic fire events can lead to the reduction of Atlantic white cedar stands if the water table at the time of ignition is below normal or the soil is too dry, causing the seed bank to burn. As a result, maple-gum and cypress-gum trees tend to repopulate these areas [27–29].

**Table 1:** Forest age classes representative by species in the Great Dismal Swamp

Forest age class	AWC [29]	CG [26]	MG [26]	PP [29]
Young	0–8	0–15	0–15	0–5
Intermediate	8–70	15–200	15–79	5–40
Mature	70–500	200–1000	80–200	40–400

*AWC* Atlantic white cedar; *CG* cypress-gum; *MG* maple-gum; *PP* pond pine

Cypress-gum (*Taxodium distichum*–*Nyssa biflora*) stands in the GDS are dominated by bald cypress and tupelo gum, [24]. Cypress-gum stands are characterized by frequent, prolonged flooding from January to June, on poorly drained soils. They are slow-growing, but long-living in comparison to the other forest communities in the GDS reaching their maximum height at 200 years [26]. Mortality can occur naturally any time after 200 years, but disturbance often shortens this life span. Cypress-gum stands have the highest above-ground C densities in the GDS [25, 30–32].

Historical maps and records show large proportions of dominant bald cypress stands within the wetter gradient of the GDS, while the current footprint covers only 12% of the swamp (Fig. 1). The sampling done in the late 1970s showed an average age of 86 years, with an average basal area growth of 0.94 m^2^/ha^−1^/year^−1^ [25]. Day [27] discusses a reduction in the abundance of bald cypress in the GDS as a result of drying conditions and logging.

Maple-gum is a forest community found in the GDS that includes red maple (*Acer rebrum*) and black gum (*Nyssa sylvatica*). Red maple, the dominant of the two, is a hardwood species that is native to a wide region in North America, ranging from Newfoundland in the north, to Florida in the south, and as far northwest as Illinois and southwest to Texas [33]. With the geographic gradient in range comes the ability to withstand the same wide variance in climate, soil type and topography. Red maple can grow in conditions ranging from dry escarpments to peat bogs. Given this high level of adaptation, and a legacy of ecosystem alteration, maple-gum has become the dominant forest community in the GDS covering approximately 61% of the landscape (Fig. 1). The life span of maple-gum is shorter than the other forest communities, reaching full maturity at 70–80 years, and rarely living beyond 150 years. Average mature trees are 18–27 m in height and 46–76 cm in DBH [34].

Pond pine (*Pinus serotina*), is a fire-adapted species that is predominantly located along the Atlantic Coastal Plain from Virginia to South Carolina [29]. Pond pine is commonly found in pocosin wetlands, which is why the forest type is often referred to as ‘pond pine pocosin’ in the GDS. Biophysical characteristics of the species vary based on peat depth, soil saturation, and fire history. Pond pine stands present in seasonally flooded swamps are stunted in growth compared to the same species growing in drier soils with good drainage. The height of pond pine increases with decreasing peat depth. An open canopy with an intermediate age of 10 years averages heights of 3 m, while a mature stand with an age of 50 years averages a height of 14 m and an average DBH of 26 cm [35]. More than 15% of the GDS is covered by pond pine (Fig. 1) and restoration efforts have been successful by removing maple-gum stands and replanting pond pine.

## Methods

### Model purpose and design

The LUCAS model was initially developed to bridge the gap between spatial allocation models capable of characterizing land-use and management actions, and process-based models for biological C cycle dynamics. To do this, the model uses a STSM model coupled with a C Stock-Flow model. This paper describes the LUCAS model development for the GDS, which annually tracks changes in four vegetation communities (Atlantic white cedar, cypress-gum, maple-gum and pond pine) and their corresponding C stocks. Below we present the parameterization of the LUCAS model to estimate C balance of the GDS ecosystem for the historic period 1985–2015.

### State variables and scales

The spatial extent of the GDS model corresponds to the current managed lands within the GDSNWR and the Dismal Swamp State Park covering about 54,000 ha. The landscape was partitioned into a spatial grid of 100 m by 100 m (1-ha) simulation cells. Simulations were run for 30 years on an annual time-step spanning the historical period 1985–2015.

Each simulation cell is defined by a unique set of state variables that characterize the landscape conditions [18]. For this model, the first state variable consists of six land-cover/vegetation types (state classes) intersected by two moisture zones (strata). The state classes coincide with the desired and undesired vegetation types central to land management: Atlantic white cedar (desired), pond pine (desired), cypress-gum (desired), maple-gum (undesired), Marsh (undesired) and Open Water. Delineation of the moisture zones (Wet and Dry) was based on interactive mapping done by local land managers, stakeholders and scientists. The second state variable is the age of each cell. All cells with state variables that are associated with forest vegetation are assigned an age (i.e. how old the tree is), which is tracked in annual time-steps for the duration of the model simulation.

**Fig. 2:**
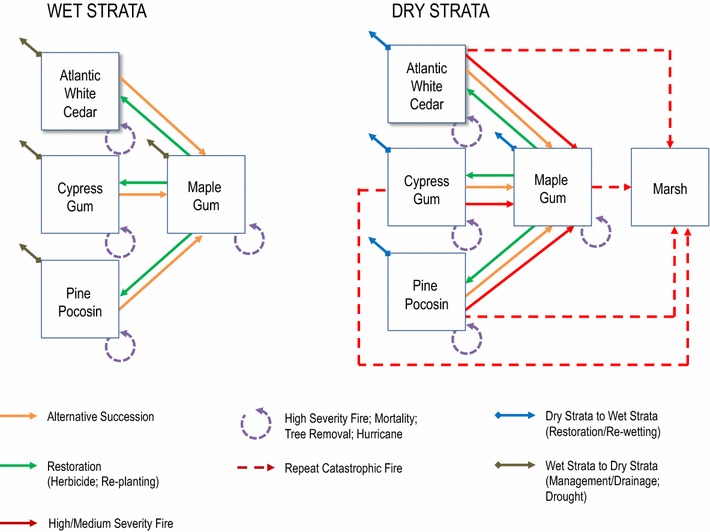
Pathway diagram for the Great Dismal Swamp state-and-transition simulation model. The* boxes* represent the state types and the* lines* with arrows represent the possible transitions. The *different colored lines* signify the different transition types. The *blue arrows* indicate a strata change from Dry to Wet due to restoration (hydrologic re-wetting). The *brown arrows* indicate a strata change from Wet to Dry due to managed draining or prolonged drought. The Dry strata represent a vulnerable system at risk of catastrophic fire and hurricane events

Transitions are used in the model to signify conversions from one state class to another within an annual time-step. Transition pathways are defined to represent all possible conversions simulated within the model including changes associated with alternative succession, wildfire, large storms, restoration, management, and changes in the size and spatial distribution of moisture strata (Fig. 2). The order in which the transition pathways are conducted is random for each time-step. Transition probabilities are assigned to each pathway and can be specified as stationary or varying. This flexibility allows one to measure the sensitivity of individual variables during calibration. Figure 2 illustrates a pathway diagram with all possible transitions between states using a box and arrow design. The boxes represent the state types and the arrows represent the possible transitions with colors and dashes representing different transition types.

### Transition probabilities

With the LUCAS model, transitions can be defined as probabilistic transitions or as discrete area targets. For this model we used transition targets to represent known events that occurred over the historical model period, including wildfires, storm damage, and management activities. The Monitoring Trends in Burn Severity (MTBS) database provided the spatial distribution, severity, and frequency of fires (i.e. patch size, location and date) for the years 1984–2014 [36]. Within the Dry strata, if two fires occurred on the same patch, within 5 years of each other, the second fire was classified as “repeat catastrophic fire”. For high severity and repeat catastrophic fires, the age of the cell was reset to zero while medium and low severity fires did not result in a reset of age. We simulated six fire events over the 30-year simulation, including the South One (in 2008) and Lateral West (in 2011; repeat catastrophic). In years with an identified fire, the transition probability for each cell within the burn perimeter was set to 1.0 and all other cells were set to zero. Fire severity within a burned area was simulated randomly based on proportions derived from the MTBS dataset (Table 2).

**Table 2:** The proportion of all fires that fall within high, medium, or low burn severity

Transition type	Proportion	Age reset
FIRE: high severity	0.163	Yes
FIRE: med severity	0.409	No
FIRE: low severity	0.428	No

These values are derived from the MTBS dataset [36]

To represent storm damage over the historical period we assumed within the South One/Lateral West perimeter, 625 ha of Atlantic white cedar was destroyed as a result of Hurricane Isabel in 2003 [37]. As a result, the age of each impacted forest cell was reset to zero. Restoration efforts in these storm-impacted areas initiated the mechanical removal of dead trees followed by replanting Atlantic white cedar seedlings. Within the model, we estimated 316 ha were treated over a 4-year period between 2004 and 2007 [37].

Alternative successional pathways (i.e. conversion from Atlantic white cedar and pond pine to maple-gum) represent the conversion from one state class type to another as a result of natural disturbance. Probabilities were derived from LANDFIRE’s biophysical settings models [29] and specified for the Atlantic white cedar and pond pine forest communities. Conversion to the maple-gum state class was made possible for state class types where the age of the cell was less than or equal to 5 years old. The rationale behind this transition parameter is that alternative succession has the highest probability of occurring if the forest stand was recently cleared. If an Atlantic white cedar stand regenerates and grows for 5 years, the stand is considered established and the probability of alternative succession to maple-gum greatly declines. Within the Dry zone, the probabilities for Atlantic white cedar and pond pine were 0.80 and 0.50, respectively. Within the Wet zone, the probabilities were 0.04 for Atlantic white cedar and 0.01 for pond pine. Additionally, adjacency rules were established to drive the spatial placement of transitions into cells immediately adjacent to existing cells classified in the “to” category. For example, transitions into maple-gum due to alternative succession can only occur in cells directly adjacent to existing maple-gum cells. The adjacency rule uses a “moving window” approach to evaluate the eight neighboring cells around it. The result gives the cell with the highest proportion of matching neighbors, the highest likelihood of transitioning. The process for calculating adjacency probabilities was updated every 5 years for all transitions.

### Carbon stock-flow model

In addition to state-and-transition modeling functions to simulate ecosystem behavior and management, the ST-Sim software offers a stock and flow module/add-on that tracks changes in C stocks and flows (i.e. fluxes) over time. To evaluate C budgets as a function of land management, the GDS project objective devises the use of all ongoing in situ field work to characterize biomass stocks and flow rates. Since this field collection is not complete to date, the baseline C budget has been developed using literature values. Table 3 shows the specific stocks and flows that are modeled as part of the baseline C budget for the GDS, as well as the data source and method by which they were derived. The stock and flow model tracks C stocks (i.e. pools or reservoirs) as a biomass measurement for each simulation cell using a gain-loss method consistent with “good-practice” recommendations from the Intergovernmental Panel on Climate Change (IPCC) [38]. Within each time-step (e.g. year), C can flow from one stock to another at specified rates, capturing annual gains and losses. The equation below shows an explanation of how the IPCC-recommended, gain-loss method is calculated.

$$\Delta {\text{C}} = \sum {\left[ {{\text{A}} * \left( {{\text{C}}_{\text{g}} - {\text{C}}_{\text{l}} } \right)} \right]}$$

where, A area of land, ha; Cg annual rate of gain (flow) of carbon, metric tons C per ha per year; Cl annual rate of loss (flow) of carbon, metric tons C per ha per year.

**Table 3:** Field collection methods for each stock and flow type

Stock and flow types	Method	Species dependent	Reference
*Carbon stocks*			
Live leaf	Diameter mass regressions	AWC, cypress-gum, maple-gum, mixed hardwood	[39]
Livewood	Diameter mass regressions	AWC, cypress-gum, maple-gum, mixed hardwood	[39]
Leaf litter	Forest floor harvest	AWC, cypress-gum, maple-gum, mixed hardwood	[40]
Deadwood	Forest floor harvest	AWC, cypress-gum, maple-gum, mixed hardwood	[40]
Live root	Pit harvest	Maple-gum	[32]
Live/dead root Ratios	Monthly core sampling (1 year)	Maple-gum	[41]
Root necromass (Dead)	50% of live root–average live/dead root ratio	Maple-gum	[41]
Soil-upper peat (0–40 cm)	Bulk density, C content, organic matter content ^a^	AWC, cypress-gum, maple-gum, mixed hardwood	This study
Soil-deep peat (41–100 cm)	Bulk density, C content, organic matter content ^a^	AWC, cypress-gum, maple-gum, pond pine	This study
*Annual carbon flows*			
Above-ground NPP	Diameter increments and regressions	AWC, cypress-gum, maple-gum, mixed hardwood	[25, 31]
Below-ground NPP	Monthly core sampling	Maple-gum	[41]
Leaf litterfall	Litter Baskets	AWC, cypress-gum, maple-gum, mixed hardwood	[42]
Tree mortality	1.5% of total Live Wood (3% on AWC)	AWC, cypress-gum, maple-gum, mixed hardwood	[43, 44]
Root mortality	Monthly core sampling	Maple-gum	[41]
Leaf litter decay	Mass loss from litter bags	Maple-gum	[27]
Deadwood decay	Mass loss from pre-weighted bole and branches	Maple-gum	[27]
Root necromass Decay	Mass loss from litter bags	AWC, cypress-gum, maple-gum, mixed hardwood	[41]
Humification to soil	Mass balance	AWC, cypress-gum, maple-gum, mixed hardwood	[43]
Soil/peat respiration—upper peat (0–40 cm)	Steady-state assumption (gain = loss)	AWC, cypress-gum, maple-gum, pond pine	This study
Soil/peat accumulation-deep peat (41–100 cm)	Long term average accumulation rate = 0.2 t C/ha^−1^year^−1^	AWC, cypress-gum, maple-gum, pond pine	[45, 46]

Stock and flow types listed here correspond to the types used for the LUCAS baseline C budget

*AWC* Atlantic white cedar; *CG* cypress-gum; *MG* maple-gum; *PP* pond pine; *NPP* Net Primary Productivity

^a^Core samples collected by U.S. Geological Survey in 1999 and 2013 and sent to Natural Resources Conservation Service Soil Survey Laboratory, Lincoln, NE

The stock and flow values used to initialize the model were derived from a body of work (see Table 3 for references) from the 1970s and 1980s, consisting of on-site field collection for the GDS forest communities of interest. The C stock types that have been identified and modeled for this application include: live wood, live leaf, live root, deadwood, leaf litter, dead root, and soil (peat). The C stock densities for each state class (forest type) that were used to initialize the model are shown in Table 4. These values represent mature stands or forest age to growth relationships that are in equilibrium. There were a few limitations related to the literature values that needed to be addressed for model development. Megonigal and Day [43] modeled four forest communities in the GDS (Atlantic white cedar, cypress-gum, maple-gum and mixed-hardwood). Carbon values for pond pine are not included in the literature we have chosen to use for this model. Although there are many individual biomass variables for pond pine from other sources [47, 48], none provide the level of thematic detail across above and below-ground categories consistent with our approach. Therefore, the stock values for pond pine shown in Table 4 are an average of Atlantic white cedar, cypress-gum and maple-gum.

**Table 4:** Initial carbon stock types and carbon density values by forest type

Stock type	AWC	CG	MG	PP^a^	Average
NPP^b^	11.35	10.78	9.29	10.47	10.47
Live leaf	5.42	3.00	2.91	3.77	3.77
Live wood	103.87	169.52	94.62	122.67	122.67
Live root	5.46	4.46	3.68	4.53	4.53
Leaf litter	5.03	4.46	4.15	4.55	4.55
Dead wood	25.07	22.70	13.38	20.38	20.38
Dead root	2.54	2.07	1.71	2.11	2.11
Upper peat (0–40 cm)	358.40	358.40	358.40	358.40	358.40
Deep peat (41–100 cm)^c^	537.60	537.60	537.60	537.60	537.60

Values for ‘Upper Peat’ and ‘Deep Peat’ are calculated with a standardized depth and soil chemistry characteristics (bulk density, organic matter and carbon content) that were measured on site. The model uses an initial peat depth of 100 cm for the entire swamp. Values are in tons carbon per hectare

*AWC* Atlantic white cedar; *CG* cypress-gum; *MG* maple-gum; *PP* pond pine

^a^Pond pine (PP) values are an average of Atlantic white cedar (AWC), cypress-gum (CG), and maple-gum (MG)

^b^NPP (net primary productivity) represented as an annual gain (t C/ha^−1^/year^−1^)

^c^Deep Peat was added as a passive carbon pool to allow the model to store carbon with long term carbon accumulation rates

### Live organic matter (live wood, live leaf, live roots)

Live organic matter consists of living vegetation above and below the soil. The literature values in Table 4 show an average live biomass value of 130.97 t C/ha^−1^, across all forest sites. When looking at the minimum and maximum by forest type, cypress-gum has the highest density at 176.98 t C/ha^−1^ and maple-gum has the lowest value at 101.21 t C/ha^−1^. Maple-gum reaches age to growth maturity around 80 years, whereas a healthy cypress-gum does not reach maturity until 200 years (Table 1), accumulating larger stocks. The lateral root standing stock (coarse and fine roots) values were derived from pit harvesting methods in the GDS with a mean estimate of 4.5 t C/ha^−1^ [32].

### Dead organic matter (deadwood, leaf litter, dead roots)

Dead organic matter in a forested ecosystem refers to non-living biomass including deadwood, litter, and root necromass (dead roots). For the LUCAS model, the deadwood stock accounts for both standing and downed deadwood. Atlantic white cedar had the highest biomass density in the literature at 25.07 t C/ha^−1^ compared to maple-gum with a value of 13.37 t C/ha^−1^. Storm blow-downs and higher mortality rates, coupled with very slow decomposition, account for the large accumulations of coarse woody debris in Atlantic white cedar stands. For the LUCAS model, leaf litter has an average density of 4.55 t C/ha^−1^. Dead roots are an important stock to account for in productive below-ground ecosystems. Based on core sampling at 1-month intervals in 1983–84 [41], dead roots are given a mean biomass density of 2.11 t C/ha^−1^.

### Soil organic matter (peat)

**Fig. 3:**
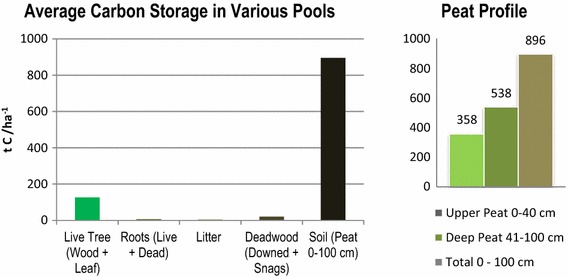
Average carbon storage by stock type. Carbon density values for both of the peat profile types used in the LUCAS model. One value is given for the Upper Peat stock, which represents 0–40 cm depth. One value is given to the Deep Peat which represents 41–100 cm depth. Carbon density values are calculated based on 100 cm depth using a bulk density of 160 cm^3^, an organic matter percentage of 95% and a C content of 59%. Values are in tons carbon per hectare

Soils in the GDS are a critical component of total ecosystem C due to the high percentage of organic matter and C content in peat, relatively low bulk density, and the substantial depths of mucky peat. The soil organic C value used to initialize the model was calculated based on bulk density and C content. Soil bulk density and carbon content were measured for 23 samples in 2013 and 3 samples in 1999 by the Natural Resources Conservation Service (NRCS) Soil Survey Laboratory in Lincoln, NE, USA [23]. Bulk density ranged from 0.09 to 0.24 g/cm^3^ and had a mean of 0.16 g/cm^3^ (160 cm^3^). Carbon content measured between 46 and 64% of total soil matter, with a mean of 59%. Organic matter averaged 95% among all samples. The U.S. Geological Survey (USGS) GDS project team is developing a spatial peat depth map, where each cell will have a unique depth; however, these data are not currently available, and so the LUCAS model used a standardized 100-cm (1-m) depth across the GDS (Fig. 3).

### Carbon flows

**Fig. 4:**
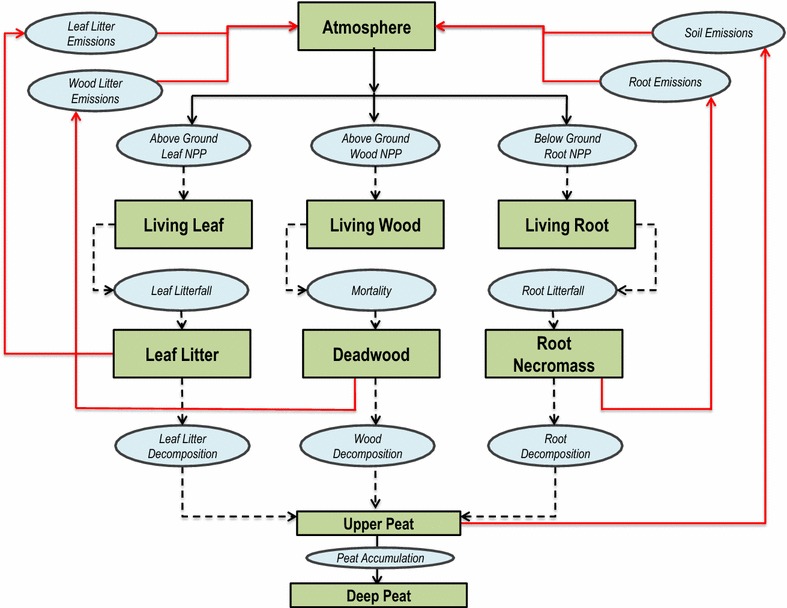
Carbon stock and flow pathway diagram for Great Dismal Swamp. *Green boxes*, Carbon stock categories; *Blue ovals*, Carbon flux categories; *Solid black lines*, NPP (input); *Solid red lines*, Emissions (output); *Dashed black lines*, Transfers between stocks

**Fig. 5:**
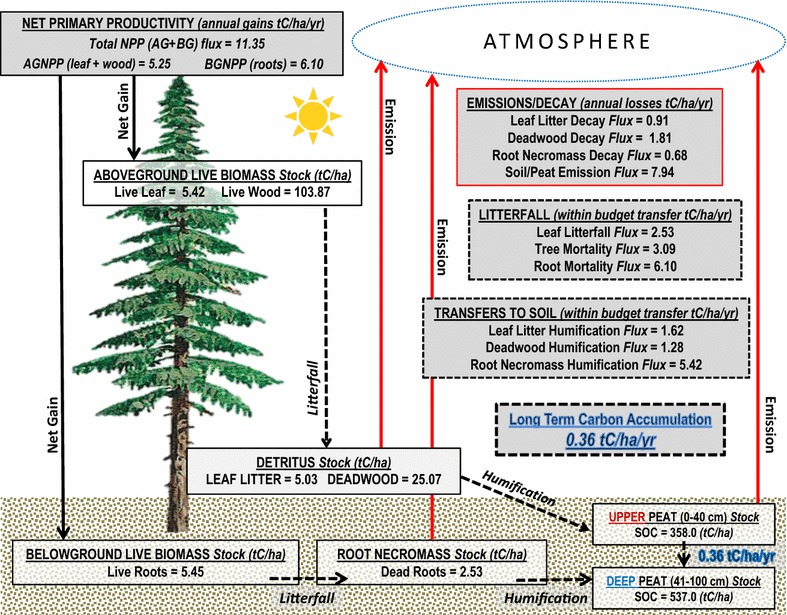
Carbon budget diagram for Atlantic white cedar in the Great Dismal Swamp

Carbon flows are the measured transfer of C from one stock to another, and are often expressed as an annual rate. The current USGS fieldwork for the GDS includes obtaining in situ flow measurements from greenhouse-gas flux towers; however, these records are a multi-year collection process and are not yet available for use as input parameters. As an alternative, C flow types and amounts reported in the literature (Tables 5, 6) have been parameterized within LUCAS to complete the baseline C budget analysis. Figure 4 shows a conceptual pathway diagram where the green rectangles represent the C stocks and the blue ovals represent the C flows. To visually demonstrate the C stock and flow amounts and how they move through an Atlantic white cedar forest, the diagram in Fig. 5 shows the annual C budget used to initialize the LUCAS model.

**Table 5:** Stock-flow pathways given as a proportional multiplier

From stock	To stock	Flow type	AWC	CG	MG	PP
Atmosphere	Living leaf	Growth:NPP	0.268	0.278	0.314	0.285
Atmosphere	Living wood	Growth:NPP	0.194	0.258	0.242	0.230
Atmosphere	Living root	Growth:NPP	0.538	0.464	0.444	0.485
Living leaf	Leaf litter	Litterfall	0.340	0.470	0.500	0.420
Living wood	Deadwood	Mortality	0.021	0.016	0.023	0.018
Living root	Dead root	Litterfall	0.528	0.528	0.528	0.528
Leaf litter	Peat	Humification	0.230	0.250	0.230	0.240
Deadwood	Peat	Humification	0.035	0.050	0.025	0.040
Dead root	Peat	Humification	0.627	0.585	0.595	0.605
Leaf litter	Atmosphere	Emission	0.180	0.176	0.229	0.185
Deadwood	Atmosphere	Emission	0.050	0.060	0.085	0.067
Dead root	Atmosphere	Emission	0.213	0.294	0.275	0.258
Upper peat	Atmosphere	Emission	0.022	0.019	0.015	0.019
Upper peat	Deep peat	Peat accumulation	0.0012	0.0015	0.0010	0.0010

In the Stock-Flow model, annual flows are expressed as a proportional multiplier of the “From Stock”. For example, when calculating Leaf Litter to Peat (Humification), the model would multiply the Leaf Litter “From Stock” value by the multiplier value (0.23)

*AWC* Atlantic white cedar; *CG* cypress-gum; *MG* maple-gum; *PP* pond pine; *NPP* net primary productivity

**Table 6:** Stock-flow pathways given as a carbon stock density in annual tons of carbon per hectare

From stock	To stock	Flow type	AWC	±	CG	±	MG	±	PP	±
Atmosphere	Living leaf	Growth:NPP	3.040	0.002	2.995	0.002	2.915	0.002	2.983	0.001
Atmosphere	Living wood	Growth:NPP	2.250	−0.048	2.785	−0.004	2.250	−0.002	2.413	−0.005
Atmosphere	Living root	Growth:NPP	6.105	0.001	4.995	0.007	4.120	0.005	5.073	0.005
Living leaf	Leaf litter	Litterfall	2.535	−0.692	2.640	−1.230	2.680	−1.225	2.618	−1.035
Living wood	Deadwood	Mortality	3.090	−0.909	2.600	0.112	1.420	0.756	2.370	−0.162
Living root	Dead root	Litterfall	6.105	−3.222	4.995	−2.640	4.120	−2.177	5.073	−2.681
Leaf litter	Peat	Humification	1.625	−0.468	1.685	−0.570	1.445	−0.491	1.585	−0.493
Deadwood	Peat	Humification	1.280	−0.403	1.150	−0.015	0.280	0.055	0.903	−0.088
Dead root	Peat	Humification	5.420	−3.827	4.135	−2.924	3.470	−2.453	4.342	−3.065
Leaf litter	Atmosphere	Emission	0.091	0.814	0.955	−0.170	1.235	−0.285	1.033	−0.191
Deadwood	Atmosphere	Emission	1.810	−0.557	1.450	−0.088	1.140	−0.003	1.467	−0.102
Dead root	Atmosphere	Emission	0.685	−0.144	0.860	−0.251	0.650	−0.180	0.732	−0.188
Upper peat	Atmosphere	Emission	7.940	−0.290	7.510	−0.810	6.260	−0.526	7.240	−0.392
Upper peat	Deep peat	Peat accum.	0.360	0.007	0.120	0.009	0.140	0.011	0.170	0.010

The ± columns signify the difference between the actual value and the value after model calibration

Annual flows are given as an annual carbon density (t C/ha^−1^yr^−1^)

*AWC* Atlantic white cedar; *CG* cypress−gum; *MG* maple-gum; *PP* pond pine; *NPP* net primary productivity

### Net primary productivity

Net primary production (NPP) is defined as the rate of live organic matter accumulation over a set time interval (e.g. annual), and is calculated as the difference between photosynthesis and autotrophic respiration within the given year. NPP operates as the primary input flow parameter for the LUCAS model. Global NPP is estimated at 60 Gt C year^−1^, which is about half of the estimated gross primary production at 120 Gt C year^−1^ [49].

In the GDS literature, above-ground NPP on periodically flooded sites ranged from 5.3–5.9 t C/ha^−1^ year^−1^, which is substantially greater than the above-ground NPP on the rarely flooded site, which had a value of 4.2 t C/ha^−1^ year^−1^ [25, 31]. In a more recent study of Atlantic white cedar in the GDS, Atkinson [50] sampled 60 mature and intermediate plots at the GDS between 1998 and 1999, obtaining values of live biomass, litterfall, and woody debris. Above-ground NPP was reported to be 5.0 t C/ha^−1^ year^−1^ on mature stands and 4.0 t C/ha^−1^ year^−1^ on intermediate stands. Below-ground NPP can have a substantial contribution to total NPP. The below-ground NPP used in the LUCAS model was 8.3, 6.9 and 3.6 t C/ha^−1^ year^−1^ for maple-gum, Atlantic white cedar and cypress-gum, respectively [41]. When a site is flooded, the anoxic environment slows or stunts the below-ground productivity, yet accelerates the growth and accumulation of above-ground biomass. In more recent field studies in the GDS, Atkinson [50] reports below-ground NPP on age-classed Atlantic white cedar sites using a microvideo camera with minirhizotrons at 45-day intervals to a depth of 64 cm. Mature sites reported the highest productivity at 3.93 t C/ha^−1^ year^−1^. The mature sites also reported the highest root mortality.

### Litterfall, mortality, and humification

Litterfall represents the annual flow between living biomass (live wood, live leaf, live roots) and dead biomass (leaf litter, deadwood, and dead roots). The GDS LUCAS model uses an annual leaf litter production ranging from 2.3 t C/ha^−1^ year^−1^ at a mixed hardwood study site to 2.7 t C/ha^−1^ year^−1^at a maple-gum site based on Gomez and Day [42]. Atkinson [50] observed a total annual litterfall in 1999 of 3.7 t C/ha^−1^ year^−1^ at the GDS-Mature site and 3.4 t C/ha^−1^ year^−1^ at the GDS-Intermediate site. In another study, leaf litter was collected at the GDS over 372 days; mature trees totaled 3.0 t C/ha^−1^ year^−1^ and intermediate trees totaled 2.4 t C/ha^−1^ year^−1^ of leaf litter. A 2014 paper [51] reported that leafy litter consists of 78–86% of the total above-ground litter production, and woody litter (branches) consists of 11–19%.

The rate at which trees and branches fall and transition from live biomass to dead biomass, also known as the mortality rate, has been estimated and converted into an annual rate for the four forest communities. The method that was used to estimate woody litter inputs comes from Waring and Schlesinger [44] and assumes the annual mortality to 1.5% of the live wood biomass stock. Atlantic white cedar has relatively high mortality in comparison, so an annual rate of 3% of the live wood biomass stock was used to calculate this flow in the LUCAS model.

According to Megonigal and Day [43], over 50% of the total organic matter that is transferred to the soil layer, comes from roots, therefore it is important to use direct measurements whenever possible. The root mortality rates used in the model make the assumption that root mortality equals that of root production (below-ground NPP); therefore, the values in Table 6 are slightly higher than the original sample of 3.5 t C/ha^−1^ year^−1^. An important assessment to make between the annual leaf litterfall rate (2.5 t C/ha^−1^yr^−1^) and the root litterfall rate, is the fact that root turnover contributes more mass to the detritus pool than leaf litterfall. These findings underline the importance of below-ground productivity in the GDS ecosystem and the potential C transfers to the soil/peat pool. The humification process converts leaf litter, deadwood, and root necromass into soil organic matter by means of microbial decomposition. See Table 5 for actual humification flow amounts used in the LUCAS model.

### Emissions

Heterotrophic respiration is a key ecosystem process that removes C from the soil layer to the atmosphere in the form of CO_2_. Respiration rates are extremely important to understand as they play a large role in the global C cycle. While structuring C budgets as a sequence of gain-loss correlations, heterotrophic respiration in soil often represents the end of the sequence, or the final amount of C removed from the terrestrial ecosystem. The literature did not provide a value for heterotrophic respiration.

Atkinson [50] calculated net peat respiration (total soil emissions–root respiration) at Atlantic white cedar stands in the GDS and a similar system in Alligator River NWR in eastern North Carolina. The GDS site revealed an annual soil respiration value of 7.1 t C/ha^−1^year^−1^, higher than the value of 4.0 t C/ha^−1^year^−1^ at Alligator River. These results indicate that lower water tables from draining cause an accelerated loss of C compared to the mature Atlantic While Cedar stand in the Alligator River, where water tables are consistently higher. Anoxic soil conditions in productive forested wetlands limit the decomposition or decay of dead organic matter, contributing to greater accumulations of detritus biomass and C sequestration [52, 53].

We divide the peat structure into two layers based on depth and hydrology. The acrotelm, or upper peat layer (0–40 cm depth), is periodically occupied by water, and has a higher yield, a higher permeability and a faster decomposition rate than the deep peat layer. The upper peat pool is where the model simulates heterotrophic respiration. The catotelm represents the deep peat layer (41–100 cm), which is permanently below the water table where microbial activity is very slow. The transfer of C from the upper peat to the deep peat layer is denoted in the model as the “Peat Carbon Accumulation” rate and is the mechanism for long-term C storage. Scientists are not certain if the GDS is a net sink or a net source of annual C. We model the upper peat at a steady state, where nearly all of the C flowing into the upper soil layer is lost to the atmosphere through respiration. We model a slight annual C sink from the upper peat to the deep peat; however, these values are a major source of uncertainty. Table 7 provides the range of literature values for peat C accumulation rates at various geographic scales. Based on this range of values from peatlands around world, we used a conservative average of 0.20 t C/ha^−1^ year^−1^ from the temperate boreal zone [45]. Through a model calibration process, we derived vegetation-specific peat C accumulation rates.

**Table 7:** Comparison of literature values for long-term carbon accumulation rates of peat

Geographic region	Peat carbon accumulation rates (t C/ha^−1^/year^−1^)	Reference
Global	0.29	[54]
Temperate boreal zone	0.20	[45]
Eastern and Western Europe	0.48	[55]
Southern Sweden	0.14–0.72	[56]
Bolivia (Andean Mountains)	0.47, 0.37	[57]
Ontario, Canada	0.13–0.31	[58]
Conterminous United States	0.71	[59]
Northeast United States	0.48	[45]
Florida	2.25	[60]
Atlantic white cedar (GDS)	0.36	This study
Cypress-gum (GDS)	0.14	This study
Maple-gum (GDS)	0.12	This study
Pond pine (GDS)	0.17	This study

Vegetation specific rates for this study are given based on a standardized peat depth of 100 cm

*GDS* Great Dismal Swamp

### Calibration

#### Forest age to biomass “spin up” simulation

**Fig. 6:**
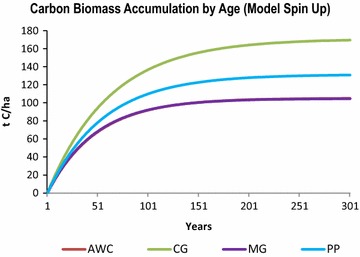
The modeled “spin up” scenario, where live-tree growth is plotted over 300 years, shows the relationship between tree age and carbon biomass for all four species. The results show that all species reach equilibrium as expected. Cypress-gum reaches equilibrium around age 200 with 160 t C/ha which is very close to the literature. Note that the Atlantic white cedar and the maple-gum growth curves overlap and reach equilibrium around 100 years around ~95 t C/ha; however, the model captures a slightly faster initial growth period for maple-gum as expected. The model spin up exercise verifies effectiveness of the carbon flow rates as parameters in the model. *AWC* Atlantic white cedar; *CG* cypress-gum; *MG* maple-gum; *PP* pond pine

Developing models that simulate complex ecosystem processes requires calibration and verification of the input variables. The C stocks we use are primarily based on mature forest stands; however, the stock-flow model runs at an annual time-step, modeling incremental growth and decay. In order to correlate the C flow rates with age-structured C stock densities, biomass growth curves are needed by species. We generated growth curves for each stock type by running a 300-year spin-up scenario in the stock-flow model. The first step in the process was to run the model in “cold-start” mode, where in the first year of the simulation all forest cells were given C stocks of zero, signifying a forest age of zero. The stocks were initialized with zero biomass, but the NPP and associated flow rates (growth, litterfall, mortality, humification and emissions) discussed in the prior section remained the same. This simulation resulted in four forest growth curves, where the growth/age equilibrium was found (Fig. 6).

In Fig. 6, cypress-gum reaches a biomass to age equilibrium at approximately 200 years old with a C biomass density of 160 t C/ha, which corresponds well with their slow growth yet long life-span. The literature indicates that this species contains more above-ground biomass per unit area, than the other species in the GDS [25, 30–32]. The model produced similar curves for Atlantic white cedar and maple-gum, both finding equilibrium at about 100 years with a C biomass of approximately 92 t C/ha. The literature indicates the mature stand age for both of these species to be about 70 or 80 years with a live tree C biomass between 85 and 105 t C/ha, depending on the health of the stand.

**Fig. 7:**
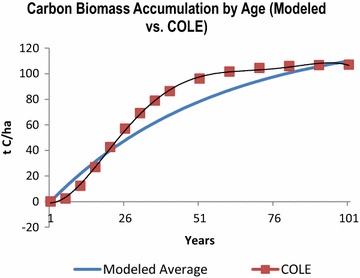
Live tree carbon biomass (by age) is shown for 100 years, where the modeled average from Fig. 6 is compared to the live tree carbon biomass by age class for the FIA plots in the Great Dismal Swamp region. The data points from the FIA were generated using COLE, a tool available from the USDA Forest Service. Both growth curves reach equilibrium at approx. 105 t C/ha, but the modeled curve lacks the signature “S” shape. The “S” shape reflects the rapid growth rate of young forests indicating a higher NPP in early growth stages. The modeled curve uses a constant NPP value. *FIA* Forest Inventory Analysis *COLE* Carbon Online Estimator

The spin-up model estimates were compared with Forest Inventory Analysis (FIA) plot-level C stocks by age class obtained from “The Carbon Online Estimator” known as COLE [61]. Because the stepped-curve from COLE gives C biomass based on all forest types within the FIA database in the GDS, the modeled growth curve in Fig. 7 represents the average from the four modeled species. The overall trajectory and range of the modeled growth curves when compared to the literature and the COLE database, confirms that the flow rates are effectively functioning at an annual time-step.

#### Forest age initial conditions map

**Fig. 8:**
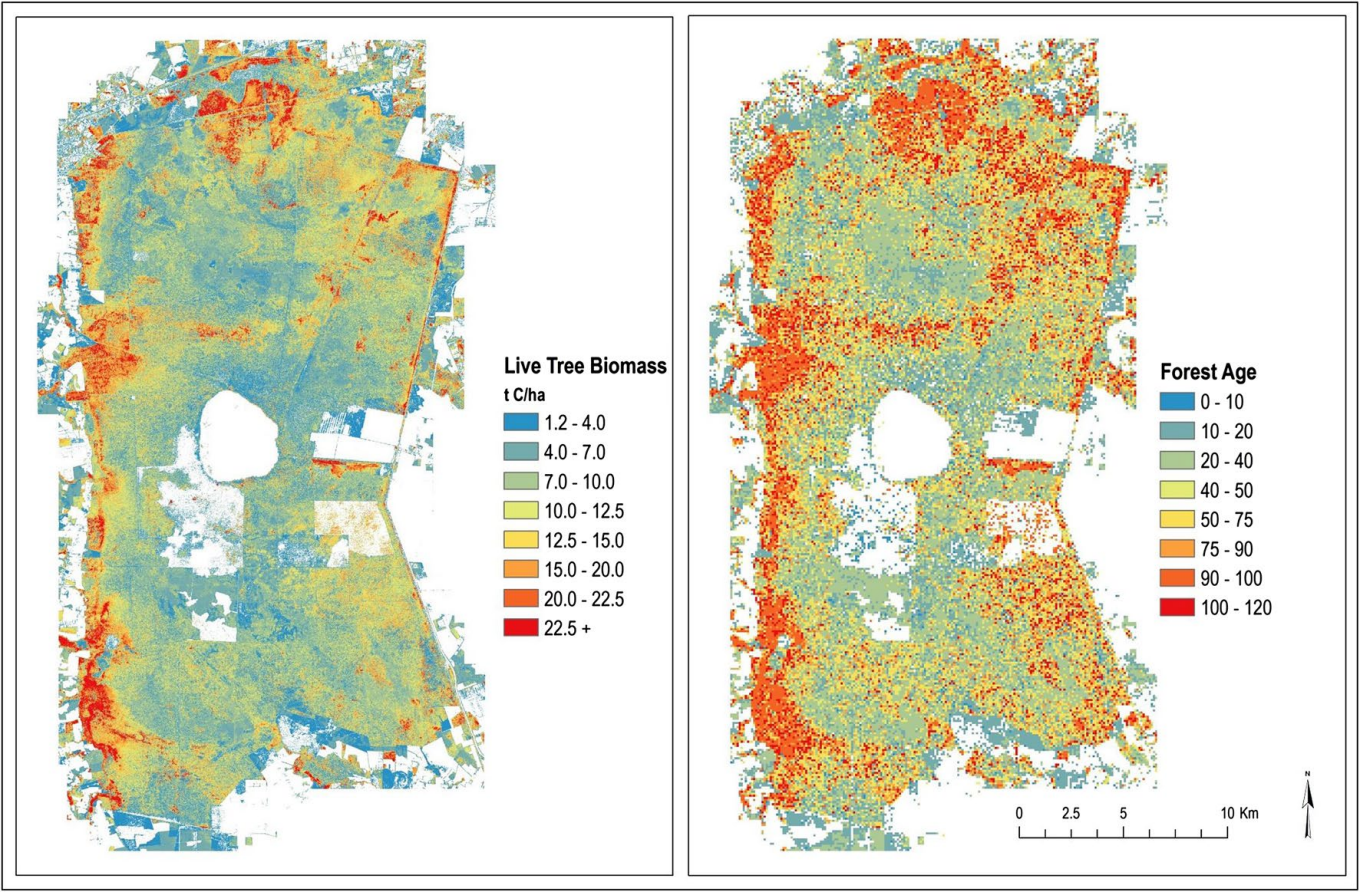
*On the left* Above-ground, live-tree carbon biomass. This map was derived from a combination of 2010 lidar and 2014 field samples (76 total plot samples) of diameter breast height by species. These two data sources were used as the variables for a linear regression model to produce wall-to-wall carbon biomass values at 10 m pixel spacing. *On the right* Forest Age map derived from the biomass map. Forest age reflects the present-day age of the above-ground biomass and does not reflect the historic age (1985). This map uses 100 m pixel spacing

In the summer of 2014, the USGS conducted an above and below-ground biomass field survey covering 76 plots, representing the four forest types modeled in the GDS project. Using a combination of the 2014 plot-level biomass measurements and 2010/2012 light detection and ranging (lidar) data, a wall-to-wall map of live-tree biomass was created with multivariate linear regression models as a technique (Fig. 8-left panel) [23]. For a complete summary of the USGS biomass field survey methods, as well as the multivariate linear regression models used, see Hawbaker et al. [23] and Hawbaker [62] respectively. The LUCAS model requires an “Age” input that can be spatial or tabular. We established a present day forest age map by using a simple look-up approach between the forest age growth curves generated from the spin-up scenario and live-tree biomass map (Fig. 8-right panel). The look-up approach used a simple conditional statement for each forested cell (e.g. if the C biomass for maple − gum = x, then the forest age for maple − gum = X).

### Historic simulation of past disturbances (1985–2015)

To test the effectiveness of the LUCAS model application for the GDS, we modeled the historic time period from 1985 to 2015, using the calibrated forest growth curves, stock and flow rates, known fire and hurricane data (spatial location, patch size, and year/severity of disturbance), and management actions related to Hurricane Isabel. Results from the model testing are compared to the recently published [23, 63] C loss estimates from the South One and Lateral West fire events.

To initialize this simulation, the present day forest age map was modified by rolling back the forest age for each cell by 30 years. We acknowledge that this is a generalization and that by simply subtracting 30 years from the current forest age, we are ignoring some of the past disturbances that may have impacted age. For the purpose of this model test, we are most interested in the area affected by Hurricane Isabel and the two catastrophic fires, which were southwest of Lake Drummond (see Fig. 1). Because most of this area had converted to marsh by the time the present day forest age map was generated, an assumption about the 1985 stand age was needed. The USFWS reported that in 2003, Hurricane Isabel uprooted and flattened approximately 1200 ha of Atlantic white cedar; which was the last pure stand in the GDS. This same general area experienced the South One and the Lateral West fires (Fig. 1). Given this information, the 1985 forest age for the disturbed area was set to 100 years, which is the average mature age for an Atlantic white cedar.

**Fig. 9:**
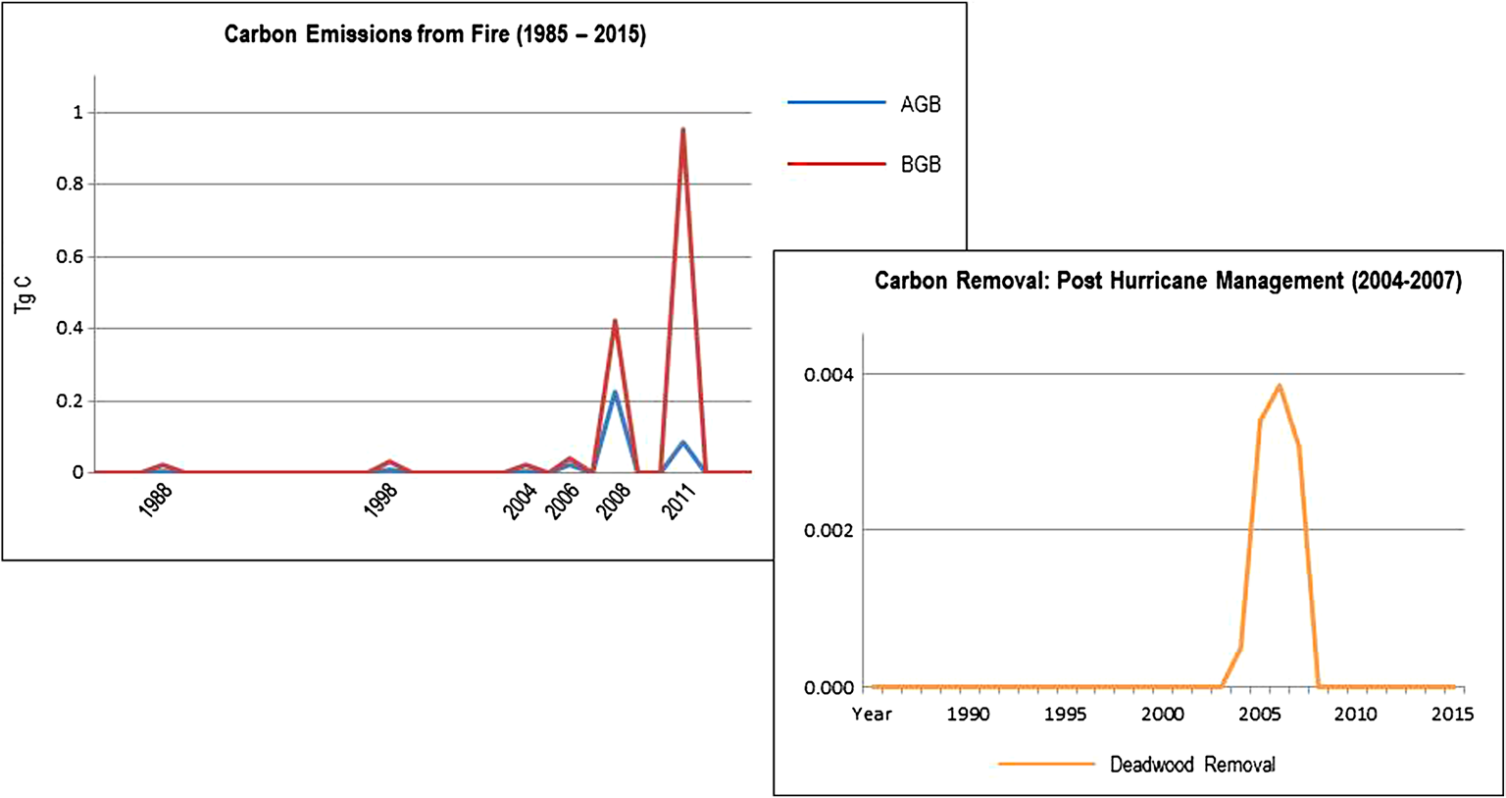
The LUCAS model captures above-ground and below-ground carbon emissions for each historic fire event. The South One fire in 2008 consumes significantly more above-ground biomass than the Lateral West fire in 2011 due to the heavy fuel loads remaining from Hurricane Isabel. When the Lateral West fire ignites, the remaining above-ground biomass was consumed as well as 0.95 Tg C from soil and roots. Carbon loss is also modeled from the management actions taken in response to Hurricane Isabel from 2003. Deadwood from approx. 300 ha of Atlantic white cedar was mechanically removed from the Great Dismal Swamp and moved to a harvested wood products pool. Values are in Tg C. AGB  Above-ground biomass; BGB Below-ground biomass

Hurricane Isabel and the six fires modeled were characterized using a spatial multiplier to show the exact location and patch size of each event. When the hurricane occurred in 2003, the model transitioned 625 ha of the live biomass to deadwood and litter. Over the next 4 years, a total transition target amount of 316 ha (781 acres) was input into the model to show the active removal of downed deadwood (Fig. 9). The deadwood was mechanically removed and salvaged for commercial use. In the model, deadwood is moved to the harvested wood products pool, at which point the C is removed from the GDS ecosystem. In the same cells where deadwood was removed, Atlantic white cedar was planted. In 2008, the South One fire burned approximately 2500 ha, of which 316 ha were newly restored from the hurricane. The South One consumed 80% of the above-ground living biomass (Fig. 9), and 50% of the upper peat layer. This amount is equivalent to 20 cm of peat depth and 0.42 Tg C (Table 8). The decision to use 20 cm of peat consumption stems from the recent work by Hawbaker et al. [23], where the 17 cm of soil elevation loss was measured with lidar.

**Table 8:** Comparison of fire emission estimates between LUCAS Reddy et al. [63] and Hawbaker et al. [23]

Results comparison	South one fire (2008)		Lateral west fire (2011)			Cumulative	
	Hawbaker et al. [23]	LUCAS Historic	Hawbaker et al. [23]	Reddy et al. [63]	LUCAS Historic	Hawbaker et al. [23]	LUCAS Historic
Below-ground carbon loss (Tg)	0.38	0.42	1.09	N/A	0.95	1.47	1.38
Above-ground carbon loss (Tg)	0.22	0.23	0.14	N/A	0.09	0.36	0.31
Deadwood removal Carbon loss from Management (Tg)	N/A	0.01	N/A	N/A	0.00	N/A	0.01
Total carbon loss (Tg)	0.60	0.66	1.23	1.10	1.04	1.83	1.70
Soil elevation loss (m)	0.17	0.20^a^	0.46	0.47	0.50^a^	0.63	0.70^a^

The latter two studies used lidar-derived elevation loss estimates pre and post fire, coupled with soil carbon characteristics to calculate carbon loss. The LUCAS model arrived at comparable results by simulating carbon gain-loss estimates between 8 pools and 14 fluxes on an annual time-step

^a^For the LUCAS model results, soil elevation loss is calculated by the soil carbon equivalent

During the 3 years between the South One and Lateral West fires, the USFWS observed Atlantic white cedar regenerating naturally, and restoration management continued by replanting saplings. With conditions already severely disturbed and dry, a lighting fire started in 2011 and burned for 144 days in approximately the same patch area. In the model, the second catastrophic fire consumed an estimated 50 cm of peat soil and the remaining above-ground biomass in the overlapping burn area. Hawbaker et al. [23] and Reddy et al. [63] measured the soil elevation loss from this fire as 46 and 47 cm, respectively (Table 8). After the repeat catastrophic fire event, the burn scar transitioned to standing water and marsh, which has started to show minimal vegetation growth, but no Atlantic white cedar regrowth.

## Results

### Net ecosystem carbon balance (1985–2015)

The net ecosystem carbon balance (NECB) refers to the long-term C storage potential of an ecosystem while factoring in the annual C gains and losses due to impacts from natural disturbance and anthropogenic land uses. Before we simulated the NECB for the GDS, we modeled the net ecosystem production (NEP) for the historic 30-year period (1985–2015). The NEP reflects the annual growth minus the heterotrophic respiration (R_h_), without factoring in disturbance or management. The NEP was estimated at an average annual rate of 0.64 t C/ha^−1^/year^−1^ (64 g C/m^2^/year^−1^) or a net sink of 0.97 Tg C.

$${\text{Growth}} - {\text{R}}_{\text{h}} = {\text{ NEP}}$$

where Growth = 14.73 Tg C; R_h_ = 13.76 Tg C; NEP = 0.97 Tg C.

When the six historic fire events were modeled during the 30-year period, including the South One and Lateral West fires, the GDS became a net source of 0.89 Tg C (NECB = − 0.89 TgC).

$${\text{Growth}} - {\text{R}}_{\text{h}} - {\text{Management}} - {\text{Fire Emissions}} = {\text{NECB}}$$

where Growth = 14.73 Tg C; R_h_ = 13.76 Tg C; Management = 0.01 Tg C; Fire Emissions = 1.86 Tg C; Fire Emissions = South One (0.66 Tg C) + Lateral West (1.04 Tg C) + Other Fires (0.16 Tg C); NECB = −0.89 Tg C.

**Fig. 10:**
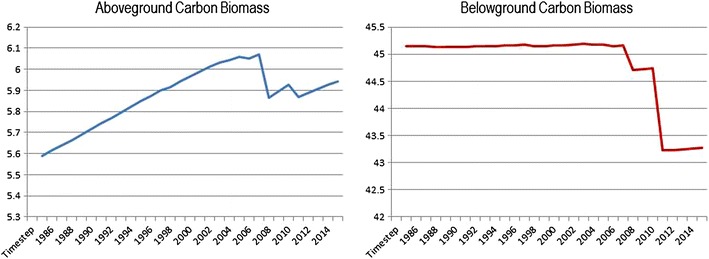
Above-ground and below-ground carbon stocks for the Great Dismal Swamp from 1985 to 2015. Above-ground stocks were aggregated for presentation purposes and include: living wood, living leaf, living root, leaf litter and deadwood. Below ground stocks include: dead root, upper peat and deep peat. Total area summarized equals 54,000 ha. Values are in Tg C

Assuming an elevation loss of 0.70 m depth for a burn scar area of 25 km^2^, cumulative above and below-ground C loss estimated from the South One and Lateral West fire events totaled 1.70 Tg C. The C loss in below-ground biomass alone totaled 1.38 Tg C, with the balance (0.31 Tg C) coming from above-ground biomass and detritus. Recent findings from Hawbaker et al. [23] estimated C losses from the South One and Lateral West fires at 1.83 Tg cumulative and 1.47 Tg below-ground. The soil surface elevation loss (0.63 m) used in these findings was derived from lidar (Table 8). In Fig. 10, the C emissions from fire events are plotted showing the difference between above-ground biomass and below-ground biomass. It is important to note that the model was able to capture the higher amount of above-ground biomass consumed in the South One fire compared to the Lateral West fire, in part due to the heavy fuel loads resultant from Hurricane Isabel. Figure 9 and Table 8 also show the amount of C that was removed from the ecosystem from management actions.

Figure 10 shows the results from all contributing C stocks during the 30-year period. The above-ground and below-ground C biomass stocks were aggregated for presentation purposes, but can also be displayed and analyzed individually and by state class (i.e. forest type) or strata (i.e. dry or wet zone). The annual C flows can also be exported and analyzed in the same way.

## Conclusion

The baseline net ecosystem C balance parameters will be used as a reference scenario in the LUCAS model for further collaboration with the GDSNWR. The overall project collaboration is designed to provide an estimate of local- and regional-scale C fluxes, ecosystem C balance, and long-term sequestration rates, with a primary goal of understanding how to maximize C sequestration on public lands while assessing potential tradeoffs with other ecosystem services. The methodology and results presented in this paper provide an operational framework for the future use of LUCAS in the GDS.

One of the strengths of our approach is the ability to isolate all of the variables on an annual basis in order to test the sensitivity of particular parameters. Although the input parameters were adapted from the literature, we used a robust calibration process and model test to validate the forest age to biomass threshold and C emissions from disturbance. As the LUCAS model for the GDS transitions to the use of in situ C stock and flow values collected by the USGS, the same calibration methods can be used to verify the forest age to biomass relationships. Preliminary results from 2014 USGS field data show a GDS average of 120 t C/ha^−1^ for above-ground biomass, which is the summed average of the live tree (leaf and wood), shrub and herbaceous stocks. The average above-ground biomass that we modeled from the literature values and provided in Table 4 is 126.44 t/C ha^−1^. The IPCC Agriculture, Forestry, and other Land Use inventory guidance [38] gives default reference values for ecological zones and forest type. For temperate continental forests, the above-ground biomass is reported at 120 t C/ha^−1^. These three above-ground biomass averages show that the preliminary plot samples, as well as the literature values used for the historic scenario in LUCAS are practical and compare well with Tier 1 IPCC guidance.

Other model inputs, however, may differ from the parameters we used to run the historic simulation. In particular, biomass pools that are typically difficult to measure in the field often use allometric equations to generate missing values. One example of this is below-ground root biomass, where many regression equations have been produced to predict live root biomass based on a ratio of the above-ground biomass [64, 65]. For example, Cairns et al. [65] summarized 160 studies of tropical, temperate and boreal forests, and found that the mean root to shoot ratio was 0.26, assuming that roots account for 26% of above-ground biomass. In a dynamic below-ground system where there has been a departure from healthy ecosystem conditions, the lack of measured root stock and growth rates will add to the overall uncertainty of future modeling.

The soil emission rates are highly variable depending on soil moisture, disturbance and biophysical conditions. The soil emission rates used in this paper are a major source of uncertainty, yet one of the most important factors for long-term C storage and accumulation. We assumed a steady-state C balance for the upper peat layer where the total C emission from soil had a net balance and did not exceed the annual C input to soil. If a higher or lower soil emission rate was used, the total ecosystem C would be greatly influenced. In situ field data are currently being collected using gas flux chambers and their use will affect the C budget modeled in LUCAS. Similarly, the long-term peat accumulation rate, where a small proportion of C flows from the upper peat layer to the deep peat layer can be a sensitive variable for total ecosystem C over a long simulation period. Due to the short 30-year time horizon used in the historic simulation, the peat accumulation rates had less of an impact on NECB; however, if a longer time horizon is modeled (i.e. 100–300 years), the rate of peat accumulation would have a greater impact on C balance.

Based on two of the driving model assumptions used in the historic simulation, a total peat consumption of 70 cm for the burn scar, and the peat accumulation rate for Atlantic white cedar of 0.36 t C/ha^−1^/year^−1^, the total soil carbon loss from the South One and Lateral West fires would take an estimated 1740 years to re-amass assuming the forest regenerated and was undisturbed. Due to the impractical time horizon this presents for land managers and decision making, this particular loss is considered permanent. Through alternative management actions such as re-wetting, below-ground biomass loss may have been avoided, resulting in the added sequestration capacity of 1.38 Tg C for the GDS. However, if the same calculation was performed using a long-term peat accumulation rate of 0.71 t C/ha^−1^/year^−1^ for the conterminous U.S. (Table 7) as conveyed in Bridgham et al. [59], the total soil C loss would take approximately 880 years to re-amass. This substantial difference points to the fact that the input parameter for the long-term accumulation rate is a highly sensitive variable and a source of uncertainty. Future model simulations will reduce uncertainty in in situ measurements of peat.

The comparison of fire emissions with the two recent studies (Table 8) show that the LUCAS model produced analogous results. These results provide a source of model validation for future applications of LUCAS in the GDS. By testing the input stock and flow parameters with past events, the model was able to capture natural processes (i.e. growth, mortality, humification, and respiration) as well as the ecosystem response to hurricane, fire and management. Land management planning often requires a stakeholder process with scenario analysis to assess future decisions. Given the robust parameter options within the LUCAS model, local and regional stakeholders can use LUCAS as an interactive decision support tool. Results benefit the evaluation of future management actions and their associated impacts on priority ecosystem services such as C sequestration, biodiversity, and disturbance prevention.

## Abbreviations

AGB: above-ground biomass
AWC: Atlantic white cedar (*Chamaecyparis thyoide*)
C: carbon
CO_2_: carbon dioxide
COLE: carbon online estimator
CG: cypress-gum (*Taxodium distichum*–*Nyssa biflora*)
BGB: below-ground biomass
DBH: diameter breast height
FIA: forest inventory analysis
GDS: great dismal swamp
GDSNWR: Great Dismal Swamp National Wildlife Refuge
GHG: greenhouse gas
IPCC: Intergovernmental Panel on Climate Change
Lidar: light detection and ranging
LUCAS: land use and carbon scenario simulator
MG: maple-gum (*Acer rubrum*–*Nyssa sylvatica*)
MTBS: monitoring trends in burn severity
NBP: net biome productivity
NECB: net ecosystem carbon balance
NEP: net ecosystem productivity
NPP: net primary productivity
NRCS: National Resources Conservation Service
PP: pond pine (*Pinus serotina*)
Rh: heterotrophic respiration
STSM: state-and-transition simulation model
USGS: United States Geological Survey
USFWS: United States Fish and Wildlife Service
